# Supplementary material for: Association between oral microbiome and seven types of cancers in East Asian population: a two-sample Mendelian randomization analysis
Source: Front Mol Biosci. 2023 Nov 21;10:1327893. doi: 10.3389/fmolb.2023.1327893 (PMC10702768; doi:10.3389/fmolb.2023.1327893)
Supplement: Supplementary file 1 [file DataSheet1.ZIP › Supplementary Materials/MR plots of saliva/saliva═╝/Pancreatic cancer/pheno.435_to_pancreatic cancer_forest.pdf]

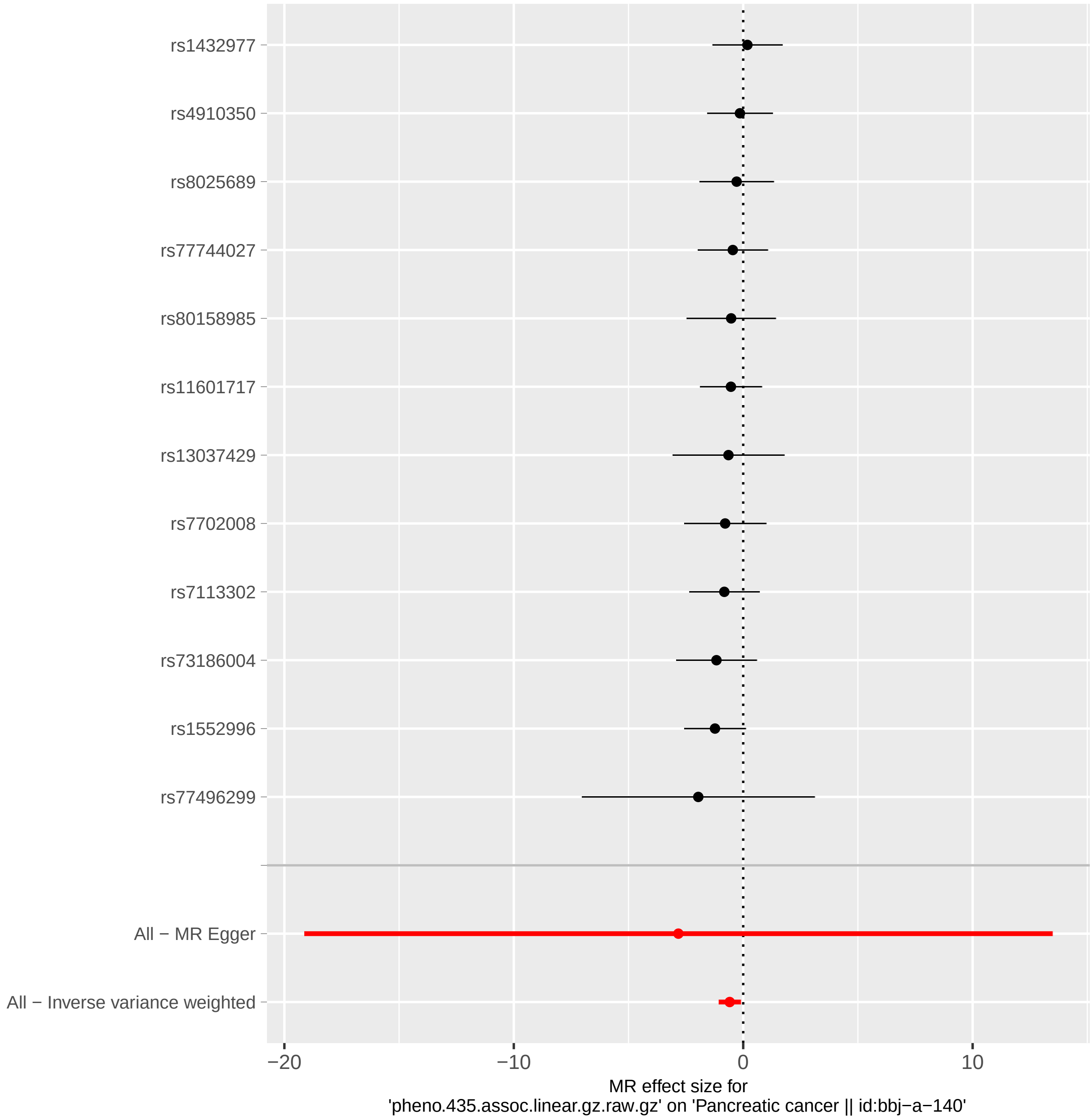
rs1432977
rs4910350
rs8025689
rs77744027
rs80158985
rs11601717
rs13037429
rs7702008
rs7113302
rs73186004
rs1552996
rs77496299
All − MR Egger
All − Inverse variance weighted
−20
−10
0
10
MR effect size for
'pheno.435.assoc.linear.gz.raw.gz' on 'Pancreatic cancer || id:bbj−a−140'
